# Isolation and Characterization of Multipotential Mesenchymal Cells from the Mouse Synovium

**DOI:** 10.1371/journal.pone.0045517

**Published:** 2012-09-18

**Authors:** Ippei Futami, Muneaki Ishijima, Haruka Kaneko, Kunikazu Tsuji, Naoki Ichikawa-Tomikawa, Ryo Sadatsuki, Takeshi Muneta, Eri Arikawa-Hirasawa, Ichiro Sekiya, Kazuo Kaneko

**Affiliations:** 1 Department of Medicine for Motor Organ, Juntendo University Graduate School of Medicine, Tokyo, Japan; 2 Department of Orthopaedics, Juntendo University School of Medicine, Tokyo, Japan; 3 Sportology Center, Juntendo University Graduate School of Medicine, Tokyo, Japan; 4 International Research Center for Molecular Science in Tooth and Bone Diseases, Global Center of Excellence Program, Tokyo Medical and Dental University, Tokyo, Japan; 5 Research Institute for Diseases of Old Age, Juntendo University Graduate School of Medicine, Tokyo, Japan; 6 Section of Orthopaedic Surgery, Graduate School, Tokyo Medical and Dental University, Tokyo, Japan; 7 Section of Cartilage Regeneration, Graduate School, Tokyo Medical and Dental University, Tokyo, Japan; University of Sao Paulo - USP, Brazil

## Abstract

The human synovium contains mesenchymal stem cells (MSCs), which are multipotential non-hematopoietic progenitor cells that can differentiate into a variety of mesenchymal lineages and they may therefore be a candidate cell source for tissue repair. However, the molecular mechanisms by which this can occur are still largely unknown. Mouse primary cell culture enables us to investigate the molecular mechanisms underlying various phenomena because it allows for relatively easy gene manipulation, which is indispensable for the molecular analysis. However, mouse synovial mesenchymal cells (SMCs) have not been established, although rabbit, cow, and rat SMCs are available, in addition to human MSCs. The aim of this study was to establish methods to harvest the synovium and to isolate and culture primary SMCs from mice. As the mouse SMCs were not able to be harvested and isolated using the same protocol for human, rat and rabbit SMCs, the protocol for humans was modified for SMCs from the Balb/c mouse knee joint. The mouse SMCs obtained showed superior proliferative potential, growth kinetics and colony formation compared to cells derived from muscle and bone marrow. They expressed PDGFRá and Sca-1 detected by flow cytometry, and showed an osteogenic, adipogenic and chondrogenic potential similar or superior to the cells derived from muscle and bone marrow by demonstrating *in vitro* osteogenesis, adipogenesis and chondrogenesis. In conclusion, we established a primary mouse synovial cell culture method. The cells derived from the mouse synovium demonstrated both the ability to proliferate and multipotentiality similar or superior to the cells derived from muscle and bone marrow.

## Introduction

Mesenchymal stem cells (MSCs) are multipotential non-hematopoietic progenitor cells that can differentiate not only *in vitro,* but also *in vivo,* into a variety of mesenchymal lineages such as osteoblasts, chondrocytes and adipocytes. Although MSCs were initially isolated from bone marrow [1], they are now able to be isolated from various types of adult mesenchymal tissue, such as the synovium, skeletal muscle, and adipose tissue, in addition to bone marrow [2], [3], [4].

**Figure 1:**
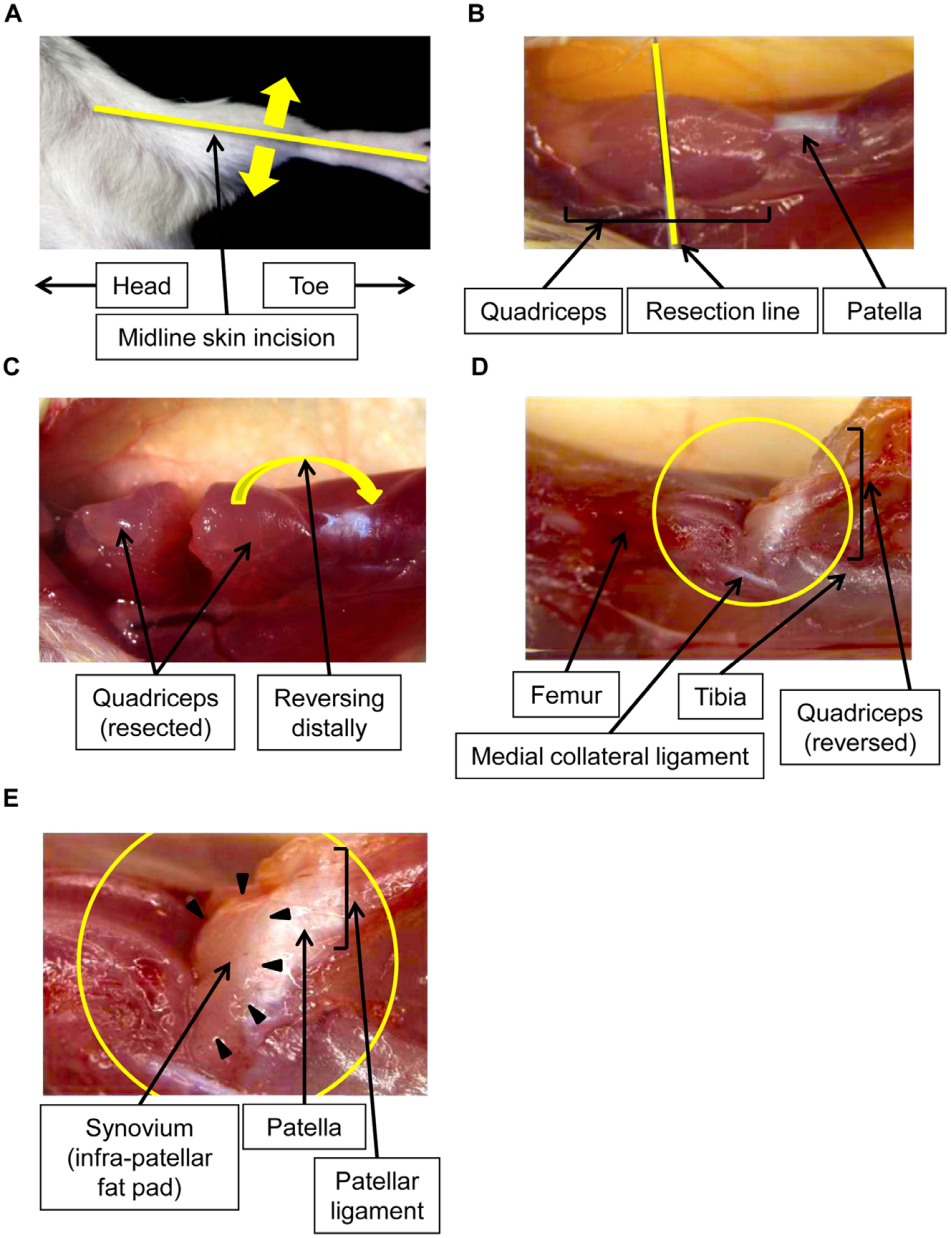
Microscopic photograph of a mouse knee to harvest the synovium. The knee joints were exposed by a midline skin incision (A). The quadriceps of the mice was microscopically resected at the middle (B) and reversed distally (C). As the femur, reversed patella and patellar ligament were exposed (D), the synovium on the infra-patellar fat pad could be easily visualized for resection purposes (E).

The synovium has a high regenerative capacity, as evidenced by its full healing after surgical and chemical synovectomy in rabbits [5], [6], [7]. The osteophytes observed at the synovium–cartilage junction in osteoarthritis are usually accompanied by excess cartilage formation [8]. When partial-thickness defects in the articular cartilage were formed in rabbits, the synovial membrane extension contributed to the repair of the cartilage [9]. Reconstructed ligaments are recovered by synovial tissue in the natural course of the healing processes [10]. All of these findings suggest that the synovium plays an important role in tissue repair in the joint.

Human synovial MSCs have a higher capacity for proliferation and greater chondrogenic potential than those from other cell sources, such as bone marrow [11]. The synovium can be collected relatively easily under the arthroscopy, while marrow aspiration is necessary for bone marrow collection. Thus, synovial MSCs are considered to be one of the appropriate candidate cell sources for tissue repair, especially for articular cartilage repair, and are now being investigated clinically as a treatment for cartilage defects [12]. Despite the impressive data reported from various investigations, there are still a lot of obstacles facing clinical research for a complete articular cartilage repair. Numerous basic research questions related to the developmental origin of these cells, their proposed pluripotency, and their molecular mechanisms of tissue repair, especially the regulation of cartilage differentiation, are also still largely unanswered [13].

Mouse primary cell culture has enabled investigators to perform research to elucidate the molecular mechanisms of the phenomena because of the relatively easy gene manipulation in such cells, which is indispensable for the molecular analysis. However, one of the obstacles we are currently confronting and have to overcome in this field is that mouse synovial MSCs have not been isolated and are not available for basic research, whereas rabbit [14], cow [15], and rat synovial MSCs [16] have been isolated, and are available for research, in addition to human synovial MSCs.

**Table 1:** The conditions for the isolation of mouse mesenchymal cells from the mouse knee joint.

	Human*/Rat**/Rabbit***	Mouse
Minced size	2–3 mm	Less than 1 mm
Collagenase concentration	0.2–0.3%	0.1%
Collagenase reaction time	1–3 hr	15 min
Deoxyribonuclease (DNase) I	−	+

* [11], **[16], ***[14].

**Table 2:** Monoclonal antibody list.

Antigen	Labeling	Clone	Isotype
CD29	PE	HMb1-1	Armenian Hamster IgG
CD34	FITC	RAM34	Rat IgG2a,k
CD44	APC	IM7	Rat IgG2b,k
CD45	APC	30-F11	Rat IgG2b,k
CD106(VCAM-1)	PE	429	Rat IgG2a,k
CD117(c-kit)	PE	2B8	Rat IgG2a,k
CD140á(PDGFRá)	APC	APA5	Rat IgG2a,k
Sca-1	FITC	D7	Rat IgG2a,k

APC: allophycocyaninFITC: Fluorescein isothiocyanate, PE: phycoerythrin.

VCAM-1: vascular cell adhesion molecule 1.

PDGFRα: platelet derived growth factor receptor alpha.

**Table 3:** Primers for specific polymerase chain reaction.

Primer	Forward	Reverse
β-actin	AGAGGGAAATCGTGCGTGAC	CAATAGTGATGACCTGGCCGT
C/EBPâ	ACCGGGTTTCGGGACTTGA	CCCGCAGGAACATCTTTAAGTGA
Col1a1	GACATGTTCAGCTTTGTGGACCTC	GGGACCCTTAGGCCATTGTGTA
Col2a1	GGGCTCCAATGATGTAGAGATG	CCCACTTACCAGTGTGTTTCG
Col10a1	GCATCTCCCAGCACCAGA	CCATGAACCAGGGTCAAGAA
FABP4	TGGGAACCTGGAAGCTTGTCTC	GAATTCCACGCCCAGTTTGA
Lpl	AGAGGCTATAGCTGGGAGCAGAAAC	GCAAGGGCTAACATTCCAGCA
PPARã	GCCCAGGCTTGCTGAACGTGAAG	CACGTGCTCTGTGACGATCTGCC
Sox9	CAGCAAGACTCTGGGCAAG	TCCACGAAGGGTCTCTTCTC
Osteocalcin	GACCATCTTTCTGCTCACTCTG	GTGATACCATAGATGCGTTTGTAG
Osteopontin	CAGTGATTTGCTTTTGCCTGTTTG	GGTCTCATCAGACTCATCCGAATG
Runx2	GCACAAACATGGCCAGATCA	AAGCCATGGTGCCCGTTAG

C/EBPâ: CCAAT/enhancer binding protein beta, FABP4: Fatty Acid Binding Protein 4, Lpl: Lipoprotein Lipase, PPARã: Peroxisome Proliferator-Activated Receptor gamma, RUNX2:Runt-related transcription factor 2.

The aim of this study was to establish a primary synovial mesenchymal cell (SMC) culture method for cells isolated from the synovium of mouse knee joints, and to characterize these cells and determine whether they can function as MSCs.

**Figure 2:**
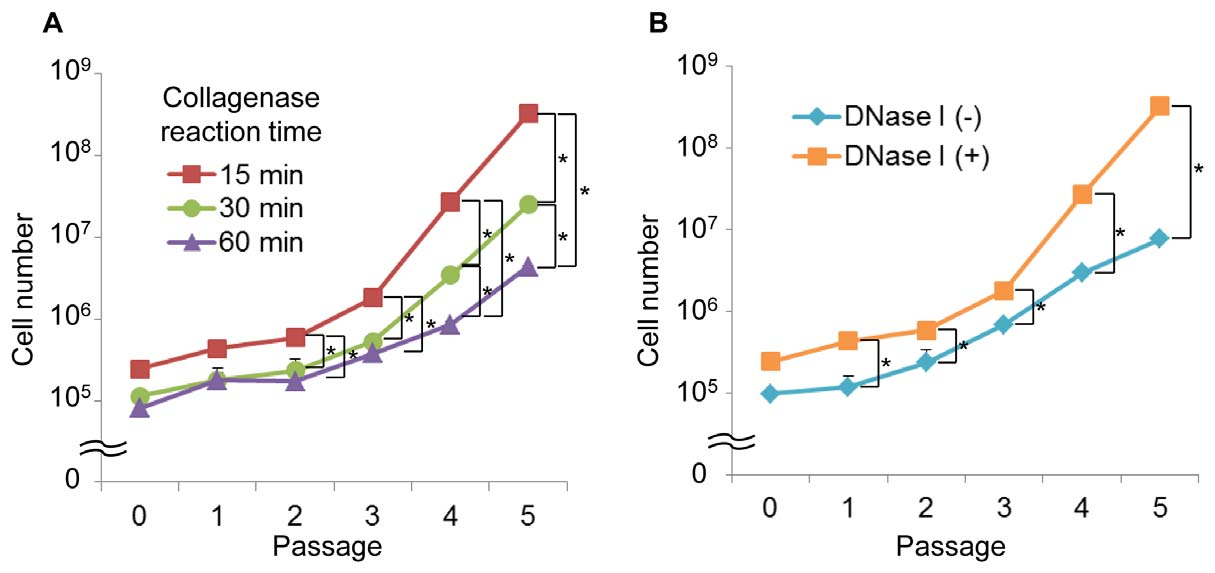
A comparison of the mouse SMC proliferation potentials following digestion by different collagenase reaction times. (A) and by the presence or absence of DNase I (B). DNase I; deoxyribonuclease I, SMC; synovial mesenchymal cell. * indicates p<0.05.

## Materials and Methods

### Tissue Collection

Ten 10-week-old female Balb/c mice were prepared for the study. The synovium in the infra-patellar fat pad of these mice was harvested [details in the Results (**Figure 1**)]. Bone marrow was flushed from the femur and tibia of these mice. Muscle was obtained from their quadriceps. The protocol of this study was approved by the Institutional Animal Care and Use Committee of Juntendo University (Registration Number: 971, Permit Number: 220084, 230017). All experimental procedures were performed following the guidelines for the care and use of animals of Juntendo University.

**Table 4:** Date on the cell samples.

	Sample size	Nucleated cell no.
Bone marrow	0.15 (0.02) (ml)	1.72×10^7^ (1.90×10^6^) (cells/ml)
Synovium	1.29 (0.16) (mg)	4.35×10^4^ (8.36×10^3^) (cells/mg)
Muscle	13.84 (4.59) (mg)	3.73×10^4^ (9.10×10^3^ ) (cells/mg)

Data (SD) (unit).

### Isolation and Culture of Mouse Cells

The mouse SMCs could not be isolated using the same protocol as that used for the isolation of human, rat and rabbit SMCs. Therefore, the protocol for human SMC culture had to be modified to obtain the appropriate conditions. The details of the methods used for the isolation and culture of mouse SMCs were also described in the Results (**Figure 1** and **Table 1**). The reagents used in this study were phosphate buffered saline (PBS), collagenase (Wako, Osaka, Japan), deoxyribonuclease I (DNase I: Sigma-Aldrich, St Louis, MO, USA) and Dulbecco’s modified Eagle’s medium (DMEM: Wako). Nucleated cells from the bone marrow were isolated with a density gradient (Ficoll-Paque; Amersham Biosciences, Uppsala, Sweden). Colony forming cells derived from muscle were used as muscle derived cells for this study. The muscle was digested in 0.1% collagenase 0.005% deoxyribonuclease I in DMEM at 37°C for 1 hour, and filtered through a 70-µm nylon filter (Becton Dickinson, Franklin Lakes, NJ, USA) The nucleated cells were plated in 6-well dishes for 24 hours in complete culture medium [DMEM containing 10% fetal bovine serum (FBS), 100 unit/mL penicillin and 100 µg/ml streptomycin (Invitrogen, Carlsbad, CA, USA)] and incubated at 37°C with 5% humidified CO_2_. The medium was changed every 3–4 days thereafter and then were cultured for 14 days as passage 0.

**Figure 3:**
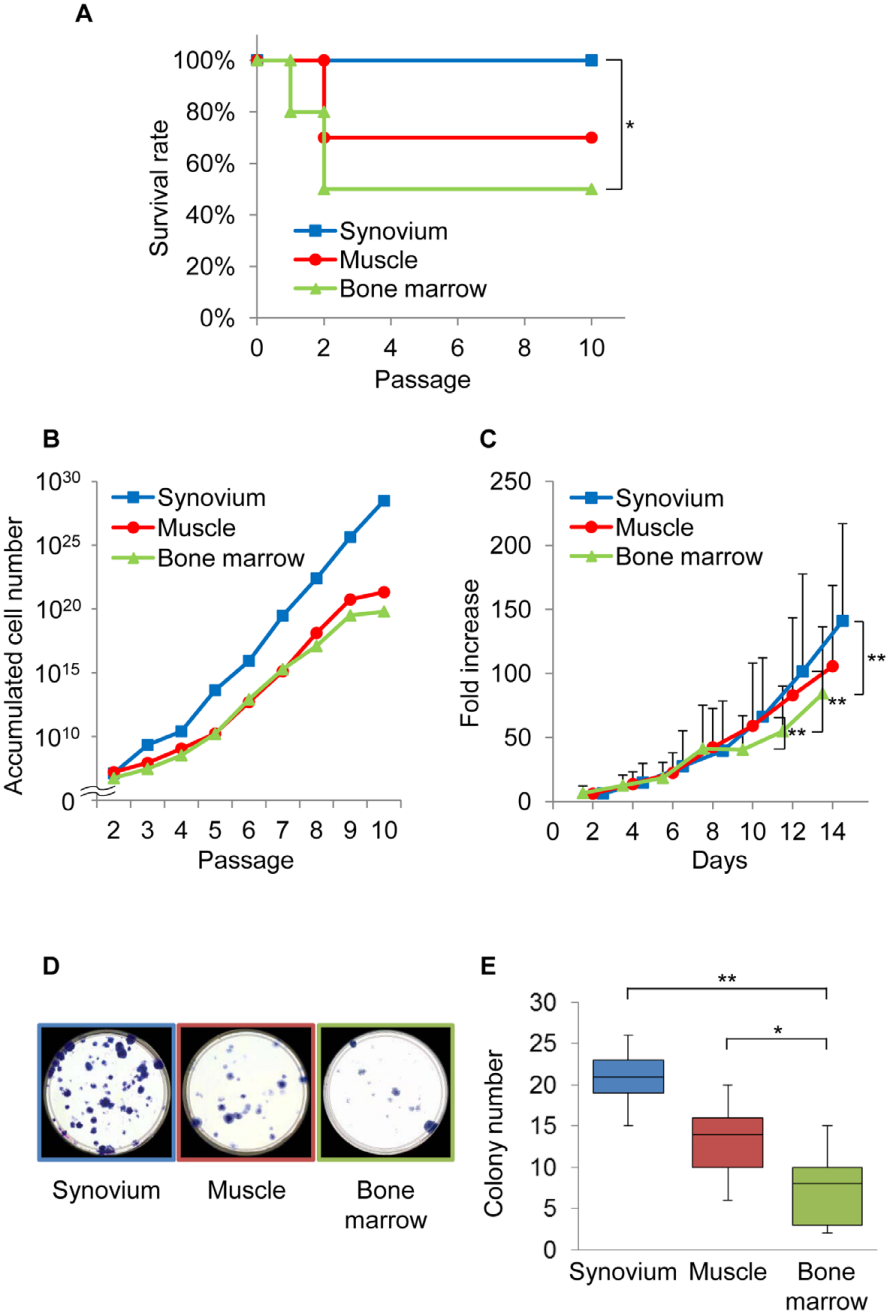
The expansion ability of mouse SMCs. (A): The survival rate of the cells. (B and C): The growth of the cells. (D): Crystal violet staining. (E): Quantification of the colony-forming ability. * and ** indicate p<0.05, and <0.01, respectively.

### Cell Expansion Assay

To compare the survival of the cells, cells derived from the synovium, muscle and bone marrow were replated every 14 days. The number of cells that were able to be replated ten times was counted. A Kaplan-Meier survival analysis was conducted to compare the survival rates of the cells.

**Figure 4:**
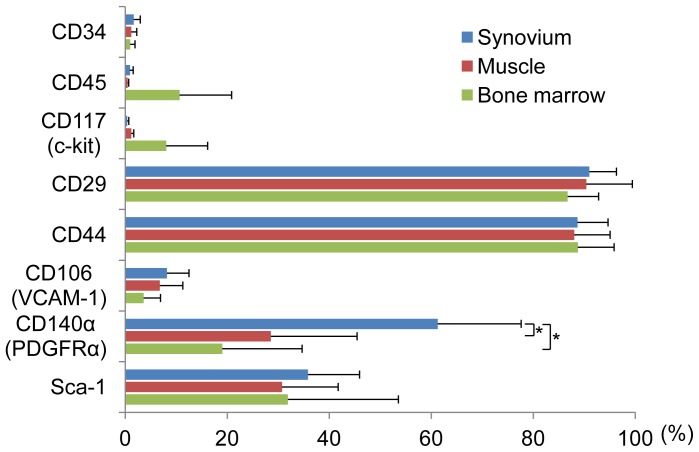
The epitopic properties of mouse SMCs. The flow cytometric analysis of mouse SMCs (blue bars), muscle–derived cells (red bars), and bone marrow derived cells (green bars). The values are the means and SD of the percent expression for each cell-surface protein. VCAM-1: vascular cell adhesion molecule 1, PDGFRα: platelet derived growth factor receptor alpha.

Nucleated cells derived from the synovium, muscle and bone marrow were clonally expanded for 14 days, and cells at passage 2 were plated at 2,000 cells/cm^2^ on 60-cm^2^ dishes. Then, the cells were replated at 2,000 cells/cm^2^ every 14 days until passage 10.

For the growth kinetics study, the cells derived from mouse synovium, muscle and bone marrow at passage 4 were plated at 1,000 cells/cm^2^ and counted with a hemocytometer every two days until 14 days.

### Colony-forming Efficacy

The cells at passage 4 were replaced at 1000 cells per 60-cm^2^ dish, incubated for 14 days, and stained with 0.5% crystal violet in 4% paraformaldehyde for 5 minutes. The cells were then washed twice with distilled water, and the number of colonies per dish was determined. Colonies less than 2 mm in diameter and faintly stained colonies were ignored.

**Figure 5:**
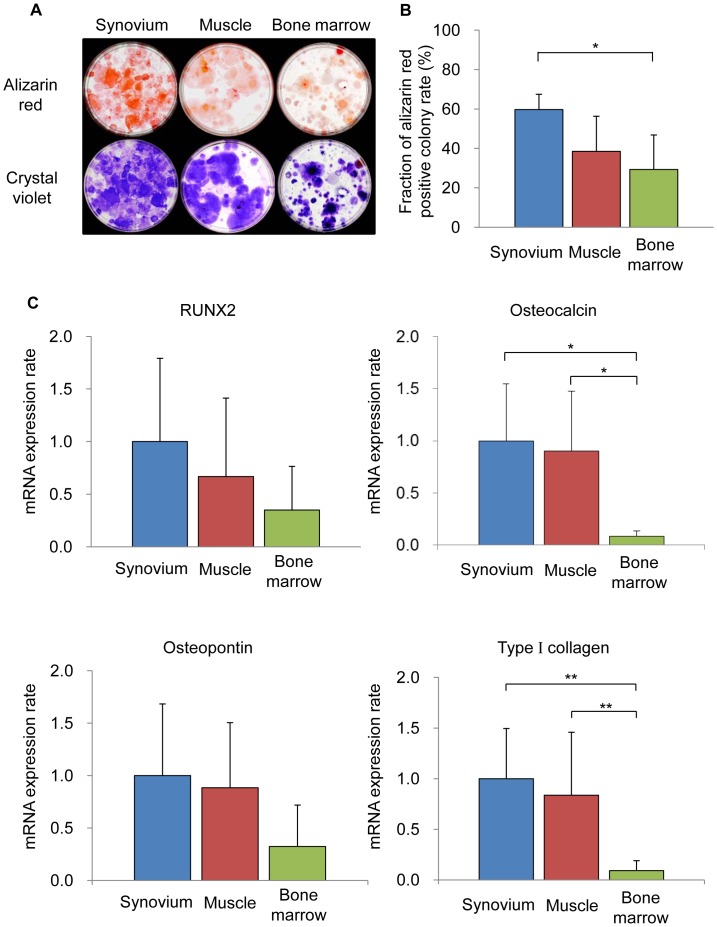
The osteogenic potential of mouse SMCs. (A): Calcified colonies stained with alizarin red (upper lane) and total colony number (lower lane). (B): The ratios of alizarin red–positive colonies to the total colonies. (C): qRT-PCR analyses for RUNX2, osteocalcin, osteopontin and type I collagen for osteogenesis.

### Flow Cytometry

One million cells at passage 3 were suspended in 500 µl PBS containing 20 µg/ml of antibody. After incubation for 30 minutes at 4°C, the cells were washed with PBS and suspended in 1 ml PBS for the analysis. Fluorescein isothiocyanate (FITC)-, phycoerythrin (PE)- or allophycocyanin (APC)- coupled antibodies against mouse CD29, CD34, CD117, and CD140á were obtained from eBioscience (San Diego, CA, USA). The CD44, CD45, CD106, and Sca-1 antibodies were from BioLegend (San Diego, CA, USA). For the isotype controls, FITC-, PE- or APC-coupled nonspecific rat and hamster IgG (eBioscience) was substituted for the primary antibody. The details of the antibodies used for flow cytometry are shown in **Table 2**. Cell fluorescence was evaluated by flow cytometry using a FACSAria instrument (Becton Dickinson, Franklin Lakes, NJ, USA), and the data were analyzed using the CellQuest software program (Becton Dickinson).

**Figure 6:**
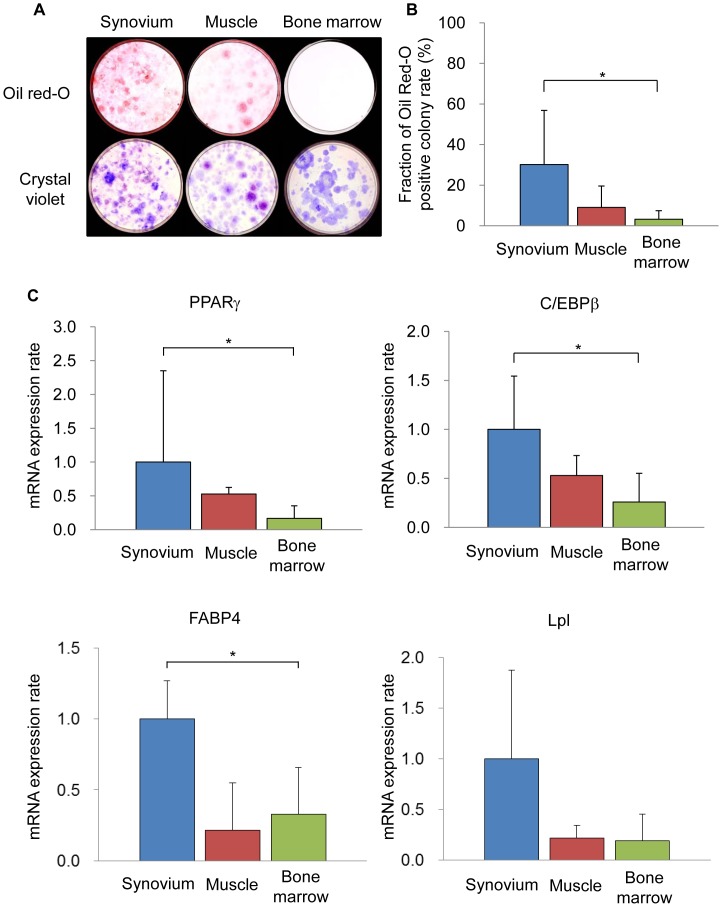
The adipogenic potential of mouse SMCs. (A): Oil red O (upper lane) and total colony number (lower lane). (B): The ratios of Oil red-O positive colonies to total colonies. (C): qRT-PCR analyses for PPARã, C/EBPâ, FABP4 and Lpl for adipogenesis.

### Osteogenesis in a Colony-forming Assay

One thousand cells were plated in 60-cm^2^ dishes and cultured in complete medium for 7 days. The medium was switched to calcification medium that consisted of complete medium supplemented with 1 nM dexamethasone (Sigma-Aldrich), 20 mM glycerol phosphate (Sigma-Aldrich), and 50 µg/ml ascorbate-2-phosphate (Sigma-Aldrich) for an additional 21 days. The osteogenic medium was replaced every 3–4 days. The dishes were subsequently stained with fresh 0.5% alizarin red solution, and the number of alizarin red–positive colonies was determined. Colonies less than 2 mm in diameter and faintly stained colonies were ignored. The same calcification cultures were then stained with crystal violet, and the total number of cell colonies was determined [11], [16].

**Figure 7:**
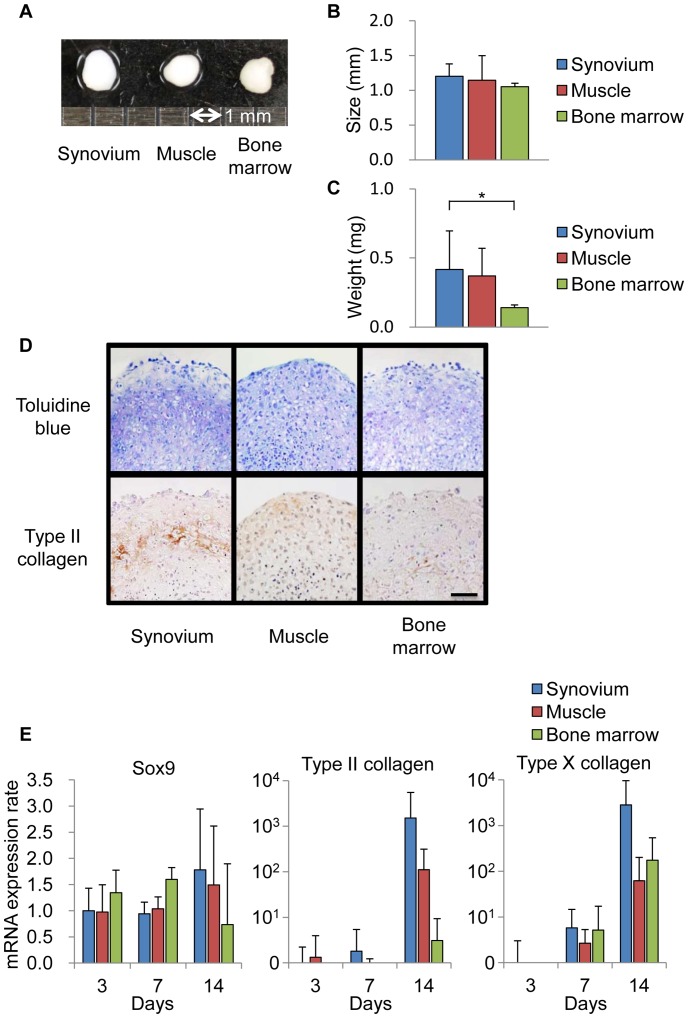
The chondrogenic potential of mouse SMCs. (A): Representative macroscopic findings of the pellets on a 1-mm scale. (B): The pellet size. (C): The wet weight of the pellets. (D): The histological features after staining with toluidine blue (upper lane) and immunohistochemistry for type II collagen (lower lane). Scale bar = 100 µm (E): qRT-PCR analyse for Sox9, type II and type X collagen.

### Adipogenesis in a Colony-forming Assay

One thousand cells were plated in 60-cm^2^ dishes and cultured in complete medium for 7 days. The medium was then switched to adipogenic medium that consisted of complete medium supplemented with 10 nM dexamethasone, 0.5 mM isobutylmethylxanthine (Sigma-Aldrich), and 50 µM indomethacin (Sigma-Aldrich) for an additional 21 days. The adipogenic medium was replaced every 3–4 days. The adipogenic cultures were fixed in 4% paraformaldehyde, stained with fresh 0.5% oil red O solution, and the number of oil red O–positive colonies was determined. Colonies less than 2 mm in diameter and faintly stained colonies were ignored. The same adipogenic cultures were subsequently stained with crystal violet, and the total number of cell colonies was determined [11], [16].

### *In vitro* Chondrogenesis

2×10^5^ cells were placed in a 15-ml polypropylene tube (BD Falcon, Franklin Lakes, NJ, USA) and centrifuged at 450×*g* for 10 minutes. The pellet was cultured at 37°C with CO_2_ in 400 µl of chondrogenic medium that contained 500 µg/ml recombinant human bone morphogenetic protein 7 (rhBMP7: Stryker biotech, Cambridge, MA, USA) in high-glucose DMEM supplemented with 10 ng/ml transforming growth factor â3 (TGFâ3; R&D Systems, Minneapolis, MN, USA), 100 nM dexamethasone, 50 µg/ml ascorbate-2-phosphate, 40 µg/ml proline (Sigma-Aldrich), 100 µg/ml pyruvate (Sigma-Aldrich), and 1∶100 diluted ITS+ Premix (Becton Dickinson). The medium was replaced every 3–4 days for 21 days. For the microscopy studies, the pellets were embedded in paraffin, cut into 5-µm sections, and stained with toluidine blue and type II collagen [17].

### Quantitative Real-time Polymerase Chain Reaction (qRT-PCR)

Total RNA from cultured cells and cultured pellets was prepared by using the Absolutely RNA Nanoprep Kit (Stratagene, La Jolla, CA, USA) according to the manufacturer’s instructions. Complementary DNA (cDNA) was synthesized using the Transcriptor High Fidelity cDNA Synthesis Kit (Roche Applied Science, Mannheim, Germany) with a random hexamer primer.

The qRT-PCR analyses of osteogenesis and adipogenesis were performed with the ABI Prism 7700 Sequence Detection System (Applied Biosystems, Foster City, CA, USA) using SYBR Green I PCR reagents (TOYOBO, Osaka, Japan) under the following condition; initial denaturation for 10 minutes at 94°C followed by 40 cycles consisting of 15 seconds at 94°C and 1 minute at 60°C. The copy number was expressed as the number of transcripts per nanogram total RNA. Experimental samples were matched to a standard curve generated by amplifying serially diluted products using the same PCR protocol. The amounts of mRNA were shown as relative quantities in comparison to that of β-actin mRNA.

To evaluate chondrogenesis, a LightCycler 480 instrument (Roche Applied Science) was used with the FastStart Taqman Probe Master (Roche Applied Science). The qRT-PCR conditions were as follows: 2 minutes at 50°C and 10 minutes at 95°C, followed by 40 cycles of 15 seconds at 95°C and 1 minute at 60°C. Normalization was performed using β-actin. Quantification was performed using the comparative Ct method [15].

For the qRT-PCR analysis, primers for the following target genes were used (**Table 3**): Runt-related transcription factor 2 (RUNX2), Osteocalcin, Osteopontin and Type I collagen for osteogenesis, Peroxisome proliferator-activated receptor ã (PPARã), CCAAT/enhancer binding protein â (C/EBPâ), Fatty acid binding protein 4 (FABP4) and Lipoproteinlipase (Lpl) for adipogenesis, Sex determining region Y-box 9 (Sox9), Type II and Type X collagen to evaluate chondrogenesis.

### Statistical Analysis

To assess significance of difference, the log rank test was used for the Kaplan-Meier survival analysis and the Mann-Whitney U test was used for other analyses. *P* values less than 0.05 were considered to be significant.

## Results

### Harvesting the Synovium from the Mouse Knee Joint

Mice were fixed in the supine and legs extended position. The knee joints were exposed by a midline skin incision (**Figure 1A**). As the mouse synovium was too small and fragile to be isolated by a common lateral approach of the knee joint, the isolation of synovium was difficult. Thus, the quadriceps reversing approach was used, as explained below. The quadriceps was transversely resected at the middle (**Figure 1B**) and reversed distally (**Figure 1C**), enabling us to distinguish between the patella and patellar ligament (**Figure 1D**). As a result, the synovium of the infra-patellar fat pad attached to the patellar ligament could easily be seen and resected from the patellar ligament (**Figure 1E**).

### Isolation and Culture of SMCs

The mouse SMCs could not be isolated using the same protocol used for the isolation of human, rat and rabbit SMCs, as shown in Table 2 [11], [14], [16], [18]. When the mouse synovium was harvested and treated according to the conditions for human, rat, and rabbit cells, the cell proliferation was poor, and the cells became flat. In most cases, the cells harvested under the conditions used for human, rat and rabbit cells were not able to be cultured even twice (data not shown). Therefore, the protocol for human SMC culture had to be modified to obtain the appropriate conditions for isolation of mouse SMCs. We determined that the collagenase concentration for the mouse synovium had to be reduced to 0.1% for the mouse from 0.2–0.3% that used for human synovium (**Table 1**). In addition, a 15-minutes collagenase reaction time (**Table 1**), which was also reduced from 1–3 hours, which is the time used for human cells [11], was determined to be appropriate for the isolation and culture of SMCs based upon the results of comparison of the cell growth (**Figure 2A**). Although DNase was not used for human cells, the supplementation with DNase I in the isolation medium showed the superior isolation and culture results for the mouse SMCs in comparison to that without DNase I (**Figure 2B**).

Based on these results, the methods used for the isolation and culture of mouse SMCs were determined as follows: The harvested synovial tissue was minced into pieces (less than 1 mm) with a surgical knife, washed thoroughly with PBS to remove hematopoietic cells, and treated with 0.1% collagenase and 0.005% DNase I in DMEM at 37°C for 15 minutes. The digested cells were filtered through a 70-µm mesh nylon filter. The quantity of the harvested tissues was measured and the isolated cells were counted using a hemocytometer (**Table 4**). The nucleated cells from the tissues were placed in 6-well dishes for 3 hours in complete culture medium and incubated at 37°C with 5% humidified CO_2_. Nonadherent cells were removed by changing the medium. The medium was changed every 3–4 days thereafter. The nucleated cells were cultured for 14 days at passage 0.

### Expansion Capacity of Mouse SMCs

To examine the characteristics of the mouse SMCs obtained by the established conditions, described above, several experiments were conducted. First, the functional capacity for self-renewal was examined. All of the mouse SMCs we obtained were able to survive until passage 10, while the mouse primary cultured cells derived from muscle and bone marrow showed survival rates of 70% and 50%, respectively (**Figure 3A**). As statistical analysis revealed that the survival rate of mouse SMCs was significantly superior in comparison to that of cells derived from bone marrow (**Figure 3A**). Next, the growth kinetics of mouse SMCs was examined. The proliferation of mouse SMCs was superior or similar to that of cells from mouse muscle and bone marrow (**Figures 3B and 3C**). The colony-forming number of mouse SMCs was significantly higher in comparison to that of mouse bone marrow and was higher than that of cells derived from muscle (**Figures 3D and 3E**).

### Epitopic Properties of Mouse SMCs

Among the eight antigens examined, the rate of positivity for CD34 (a hematopoietic progenitor cell antigen), CD45 (a hematopoietic cell marker), and CD 117 (a stem cell factor receptor) in SMCs was less than 2% (**Figure 4**). The positive ratios for CD29 and CD44 in the SMCs and cells derived from muscle and bone marrow were 90% or over. The positive ratio of CD106 (VCAM-1) in SMCs and cells derived from muscle and bone marrow was less 10%. The positive ratio of CD140á (PDGFRá) in SMCs was 50% or over, which was significantly higher than that in the cells derived from both muscle and bone marrow. The Sca-1 (mesenchymal stem cell maker) positive ratios in SMCs and cells derived from muscle and bone marrow were all approximately 30%, and no significant differences in the positivity were observed between the three cell types (**Figure 4**).

### Differentiation Potential of Mouse SMCs

#### Osteogenesis

To evaluate the osteogenic potential of the SMC populations, cells were cultured in osteogenic medium. All cells were calcified and positive for alizarin red staining (**Figure 5A**). The ratio of alizarin red-positive colonies in the SMCs was significantly increased in comparison to that in cells derived from bone marrow and was higher than that in cells derived from muscle (**Figures 5A and 5B**). qRT-PCR showed the expression levels of osteocalcin and type I collagen in SMCs and cells derived from muscle to significantly increase in comparison to those in cells derived from bone marrow. The expression levels of the mRNA for RUNX2 and osteopontin in SMCs were also higher than those in the cells derived from muscle and bone marrow (**Figure 5C**).

#### Adipogenesis

The adipogenic potential of the cells in the three populations was also compared. Lipid vesicles were observed in both SMCs and cells derived from muscle, and were less observed in cells derived from bone marrow (**Figure 6A**). The oil red-O positive colony rate, which was calculated by dividing the number of oil red-O positive colonies (**Figure 6A**, upper lane) by the number of total colonies of the same culture (**Figure 6A**, lower lane) was determined. The oil red-O positive colony rate in the SMCs was significantly increased in comparison to that in cells derived from bone marrow and was also higher than that in the cells derived from muscle (**Figure 6B**). qRT-PCR demonstrated the expression levels of PPARã, C/EBPâ and FABP4 in SMCs to significantly increase in comparison to those in cells derived from bone marrow and higher than those in cells derived from muscle. The expression levels of the mRNA for Lpl in SMCs were also higher than those in the cells derived from muscle and bone marrow (**Figure 6C**).

#### Chondrogenesis

The *in vitro* chondrogenesis pellet culture was performed to evaluate the chondrogenic potential of three cell populations. During the *in vitro* chondrogenesis, the pellet increased in size and weight, which was attributable to the production of extracellular matrix [11]. The pellet from all three cell populations became spherical after 21 days of culture (**Figure 7A**). While the size of the pellets in these three populations was similar (**Figure 7B**), the weight of the pellet from SMCs was significantly heavier in comparison to that from the cells derived from bone marrow (**Figure 7C**). The pellets from SMCs and cells derived from muscle and bone marrow consisted of extensive cartilage matrix (**Figure 7D**). A subsequent qRT-PCR study demonstrated that the pellets from all three populations expressed Sox9, type II and type X collagen, and their expression levels increased time-dependently (**Figure 7E**).

## Discussion

In this study, we determined the appropriate conditions for the isolation of SMCs from mouse knee joints (**Table 2**, **Figures 1** and **2**). The cells derived from mouse synovium demonstrated a capacity for self-proliferation (**Figure 3**) and multipotentiality (**Figures 5** **–** **7**), both characteristics of MSCs.

There have been several reports in which synovial cells were isolated from mouse arthritic joints and cultured for sebsequent experiments [19], [20]. There were also previous studies in which synovial cells were isolated from normal mouse knee joints [21], [22], [23]. However, these studies did not examine and/or describe an appropriate condition for the isolation and culture of mouse synovial cells, and furthermore they also did not examine their multipotentiality as MSCs. The current study is the first report that elucidated the appropriate conditions for the isolation and culture of mouse synovial cells, and demonstrated their proliferative capacity and multipotentiality.

The synovium is a thin layer of tissue that lines the joint space and covers a subsynovium [24]. Depending on its anatomical position, the subsynovium comprises either a fibrous or an adipose synovium, the latter is commonly called the infra-patellar fat pad. In humans, fibrous synovium-derived MSCs, which were harvested from the inner side of the joint capsule overlaying the non-cartilaginous area of the femoral condyle, and adipose synovium- (infra-patellar fat pad) derived MSCs were similar in terms of their cell morphological features, epitope profiles, colony-forming efficacy, chondrogenic, osteogenic, and adipogenic potential [25]. In this study, we microscopically isolated the mouse synovium from the infra-patellar fat pad, as isolation of the synovium from the infra-patellar fat pad is relatively easy in comparison to that from the joint capsule in mice.

The chondrogenic potential of synovial cells derived from rabbit synovium was initially reported in the 1990’s [26], [27]. In 2001, it was revealed that human synovial cells contained MSCs, which showed multipotentiality for bone, adipose tissue and cartilage [2]. Since then, these conditions for the isolation of human synovial cells have been used to isolate the mesenchymal cells of the synovium from rats and rabbits [14], [16]. However, it was found in this study that these conditions cannot be used for isolating mouse synovial cells. This may, at least in part, be due to the cytotoxicity of the collagenase used for the isolation of synovial cells that differs between humans, rats, rabbits and mouse cells, as both the collagenase reaction time and concentration had to be reduced (**Table 2** and **Figure 2**) [28].

Human synovial cells containing MSCs can be cultured for more than 10 passages [2]. The mouse SMCs in this study also showed similar self-duplicating ability (**Figure 3**). This indicates that the mouse SMCs isolated under the conditions established in this study contain MSCs similar to human SMCs.

In comparison to bone marrow- and muscle-derived cells, the synovium-derived cells showed better proliferation potential in this study (**Figure 3**). Similar observations have been made for cells derived from the synovium, bone marrow, and muscle of both humans and rats. However, care should be exercised in interpreting this findings, because the passage number of cells in which the CFU assay was conducted in this study (passage 4 or 5) was different from that of previous studies (passage 1 to 3) [11], [16]. This was because the number of colonies that formed was too small to carry out effective comparisons at passage 1 to 3 in mice, so this assay was conducted using cells at passage 4 or 5 (**Figures 3D and 3E**).

The involvement of MSCs in mouse bone marrow in proliferation was first reported in 1976 [29]. On the other hand, it is believed that although the isolation and expansion of human bone marrow cells is relatively easy [30], rodent bone marrow is difficult to expand [29], [31], [32]. The current study revealed that the viability and expandability of mouse SMCs were superior to those of mouse bone marrow cells (Figure 3). This suggests that mouse SMCs could provide not only for a cell source of MSCs, but also for elucidating the molecular mechanisms underlying the regeneration and differentiation of MSCs.

Muscle-derived cells, including in a cell line (C2C12) and primary cultured cells, have been shown to have multipotentiality [33], [34], [35], [36]. While there are several isolation methods used to obtain cells from muscle, we used the simple plate culture technique [11], [16], [37]. Muscle satellite cells are deeply associated with muscle stem or progenitor cells and actually showed multipotentiality in previous studies [3], [38], [39]. However, it is necessary to use flow cytometry to isolate these satellite cells [40], [41], [42], and they are present at a very low frequency in whole muscle cells, making their isolation difficult even when using flow cytometry for selection [43], [44], [45]. In addition, the use of flow cytometry is associated with a risk for contamination. Thus, it is not feasible to use muscle satellite cells for clinical applications.

There has been no definitive consensus about the expression patterns of the surface antigens of mouse MSCs. However, the expression patterns of mouse MSCs observed in this study were similar to those of mouse bone marrow [46], [47], [48]. The PDGFRá positive cells in murine MSCs showed superior proliferative potency and differentiation ability [46]. Similarly, the PDGFRá expression frequency in SMCs was significantly higher than that in muscle-derived cells and bone marrow-derived cells in mice (**Figure 4**). The frequency of Sca-1 positivity, which is one of the markers for MSCs, for synovium-, muscle- and bone marrow-derived cells were all approximately 30%, and no significant differences in the Sca-1 positive frequency were observed between them (**Figure 4**). This may be explained by fact that the Sca-1 positive frequency in Balb/c mice was approximately 30%, while that in NMRI mouse bone marrow cells was 50–60% [46], [48].

The osteogenetic potential of mouse SMCs was significantly superior compared to that of mouse bone marrow (**Figures 5A and B**). This was similar to that of human SMCs [11]. Consistent with this result, the RUNX2 expression of mouse SMCs was higher, but not significantly higher, than that of mouse bone marrow (**Figure 5C**). This result was also similar to that in human SMCs [11].

The adipogenic potential of mouse SMCs was significantly greater compared to that of mouse bone marrow (**Figures 6A and 6B**). In both humans and rats, the SMCs also showed superior adipogenic potential. This can be explained by the fact that both PPARã and C/EBPâ expression in mouse SMCs were significantly increased in comparison to the levels in mouse bone marrow (**Figure 6C**). These results regarding the adipogenic potential of mouse SMCs are consistent with previous studies using human and rat cells [11], [16].

In this study, the chondrogenetic potential of the cells was examined by a pellet culture system (**Figure 7**). Better extracellular matrix production was generally observed in cells derived from the human synovium by the pellet culture system. Potent cartilage matrix formation was observed in the mice in this study, however, it was not as prominent as that observed in human SMCs (**Figure 7A–D**) [11]. With regard to the mRNA expression levels of the genes encoding Sox9, type II and type X collagen, which have a crucial role inchondrogenesis on days 7 and 14, the mouse SMCs showed higher expression levels of these genes in comparison to those of mouse muscle- and bone marrow-derived cells (**Figure 7E**). From these results, we concluded that the mouse SMCs that we obtained have chondrogenic potential similar or superior to that of cells derived from mouse muscle and bone marrow.

Tissue engineering techniques using MSCs have been investigated as new treatments for tissue repair [49]. While the synovium is thought to be an appropriate cell sources for tissue engineering [50], [51], [52], [53], the molecular mechanisms are largely unknown. The mouse SMCs harvested by the established method in this study are expected to enable us to analyze the complex network of signaling pathways that regulates the proliferative and differentiation potential of synovial MSCs by conducting both *in vivo* and *in vitro* analyses of genetically modified experimental models.

In conclusion, primary mouse SMCs culture method was established by determining the conditions for isolation of the cells. The cells derived from mouse synovium demonstrated both the ability to proliferate and multipotentiality similar or superior to the cells derived from muscle and bone marrow.

## References

[1] Pittenger MF, Mackay AM, Beck SC, Jaiswal RK, Douglas R, et al (1999) Multilineage potential of adult human mesenchymal stem cells. Science 284: 143–147. 10102814 10.1126/science.284.5411.143

[2] De Bari C, Dell’Accio F, Tylzanowski P, Luyten FP (2001) Multipotent mesenchymal stem cells from adult human synovial membrane. Arthritis Rheum 44: 1928–1942. 11508446 10.1002/1529-0131(200108)44:8<1928::AID-ART331>3.0.CO;2-P

[3] Asakura A, Komaki M, Rudnicki M (2001) Muscle satellite cells are multipotential stem cells that exhibit myogenic, osteogenic, and adipogenic differentiation. Differentiation 68: 245–253. 11776477 10.1046/j.1432-0436.2001.680412.x

[4] Zuk PA, Zhu M, Ashjian P, De Ugarte DA, Huang JI, et al (2002) Human adipose tissue is a source of multipotent stem cells. Mol Biol Cell 13: 4279–4295. 12475952 10.1091/mbc.E02-02-0105 PMC138633

[5] Bentley G, Kreutner A, Ferguson AB (1975) Synovial regeneration and articular cartilage changes after synovectomy in normal and steroid-treated rabbits. J Bone Joint Surg Br 57: 454–462. 1194312

[6] Mitchell N, Blackwell P (1968) The electron microscopy of regenerating synovium after subtotal synovectomy in rabbits. J Bone Joint Surg Am 50: 675–686. 5658554 10.2106/00004623-196850040-00003

[7] Campbell WG Jr, Callahan BC (1971) Regeneration of synovium of rabbit knees after total chemical synovectomy by ingrowth of connective tissue-forming elements from adjacent bone. A light and electron microscopic study. Lab Invest 24: 404–422. 4253433

[8] Hashimoto S, Creighton-Achermann L, Takahashi K, Amiel D, Coutts RD, et al (2002) Development and regulation of osteophyte formation during experimental osteoarthritis. Osteoarthritis Cartilage 10: 180–187. 11869078 10.1053/joca.2001.0505

[9] Hunziker EB, Rosenberg LC (1996) Repair of partial-thickness defects in articular cartilage: cell recruitment from the synovial membrane. J Bone Joint Surg Am 78: 721–733. 8642029 10.2106/00004623-199605000-00012

[10] Ahn JH, Yoo JC, Yang HS, Kim JH, Wang JH (2007) Second-look arthroscopic findings of 208 patients after ACL reconstruction. Knee Surg Sports Traumatol Arthrosc 15: 242–248. 17028869 10.1007/s00167-006-0177-8

[11] Sakaguchi Y, Sekiya I, Yagishita K, Muneta T (2005) Comparison of human stem cells derived from various mesenchymal tissues: superiority of synovium as a cell source. Arthritis Rheum 52: 2521–2529. 16052568 10.1002/art.21212

[12] Koga H, Shimaya M, Muneta T, Nimura A, Morito T, et al (2008) Local adherent technique for transplanting mesenchymal stem cells as a potential treatment of cartilage defect. Arthritis Res Ther 10: R84. 18664254 10.1186/ar2460 PMC2575632

[13] Prockop DJ (2009) Repair of tissues by adult stem/progenitor cells (MSCs): controversies, myths, and changing paradigms. Mol Ther 17: 939–946. 19337235 10.1038/mt.2009.62 PMC2835176

[14] Koga H, Muneta T, Nagase T, Nimura A, Ju YJ, et al (2008) Comparison of mesenchymal tissues-derived stem cells for in vivo chondrogenesis: suitable conditions for cell therapy of cartilage defects in rabbit. Cell Tissue Res 333: 207–215. 18560897 10.1007/s00441-008-0633-5

[15] Shintani N, Hunziker EB (2007) Chondrogenic differentiation of bovine synovium: bone morphogenetic proteins 2 and 7 and transforming growth factor beta1 induce the formation of different types of cartilaginous tissue. Arthritis Rheum 56: 1869–1879. 17530715 10.1002/art.22701

[16] Yoshimura H, Muneta T, Nimura A, Yokoyama A, Koga H, et al (2007) Comparison of rat mesenchymal stem cells derived from bone marrow, synovium, periosteum, adipose tissue, and muscle. Cell Tissue Res 327: 449–462. 17053900 10.1007/s00441-006-0308-z

[17] Sekiya I, Colter DC, Prockop DJ (2001) BMP-6 enhances chondrogenesis in a subpopulation of human marrow stromal cells. Biochem Biophys Res Commun 284: 411–418. 11394894 10.1006/bbrc.2001.4898

[18] Sakamoto Y, Ishijima M, Kaneko H, Kurebayashi N, Ichikawa N, et al. (2010) Distinct mechanosensitive Ca(2+) influx mechanisms in human primary synovial fibroblasts. J Orthop Res. 10.1002/jor.21080 20108315

[19] Dulos J, Verbraak E, Bagchus WM, Boots AM, Kaptein A (2004) Severity of murine collagen-induced arthritis correlates with increased CYP7B activity: enhancement of dehydroepiandrosterone metabolism by interleukin-1beta. Arthritis Rheum 50: 3346–3353. 15476247 10.1002/art.20509

[20] Waldburger JM, Palmer G, Seemayer C, Lamacchia C, Finckh A, et al (2011) Autoimmunity and inflammation are independent of class II transactivator type PIV-dependent class II major histocompatibility complex expression in peripheral tissues during collagen-induced arthritis. Arthritis Rheum 63: 3354–3363. 21739421 10.1002/art.30522

[21] Langdon C, Kerr C, Hassen M, Hara T, Arsenault AL, et al (2000) Murine oncostatin M stimulates mouse synovial fibroblasts in vitro and induces inflammation and destruction in mouse joints in vivo. Am J Pathol 157: 1187–1196. 11021823 10.1016/S0002-9440(10)64634-2 PMC1850181

[22] Li P, Sanz I, O’Keefe RJ, Schwarz EM (2000) NF-kappa B regulates VCAM-1 expression on fibroblast-like synoviocytes. J Immunol 164: 5990–5997. 10820282 10.4049/jimmunol.164.11.5990

[23] Rhee DK, Marcelino J, Baker M, Gong Y, Smits P, et al (2005) The secreted glycoprotein lubricin protects cartilage surfaces and inhibits synovial cell overgrowth. J Clin Invest 115: 622–631. 15719068 10.1172/JCI200522263 PMC548698

[24] Mahadevan V (2008) Pelvic girdle and lowerr limb. In: Standring S, editor. Gray’s anatomy : the anatomical basis of clinical practice. 40th ed. Philadelphia: Elsevier. 1327–1464.

[25] Mochizuki T, Muneta T, Sakaguchi Y, Nimura A, Yokoyama A, et al (2006) Higher chondrogenic potential of fibrous synovium- and adipose synovium-derived cells compared with subcutaneous fat-derived cells: distinguishing properties of mesenchymal stem cells in humans. Arthritis Rheum 54: 843–853. 16508965 10.1002/art.21651

[26] Iwata H, Ono S, Sato K, Sato T, Kawamura M (1993) Bone morphogenetic protein-induced muscle- and synovium-derived cartilage differentiation in vitro. Clin Orthop Relat Res: 295–300. 8222441

[27] Nishimura K, Solchaga LA, Caplan AI, Yoo JU, Goldberg VM, et al (1999) Chondroprogenitor cells of synovial tissue. Arthritis Rheum 42: 2631–2637. 10616011 10.1002/1529-0131(199912)42:12<2631::AID-ANR18>3.0.CO;2-H

[28] Waymouth C (1974) To disaggregate or not to disaggregate injury and cell disaggregation, transient or permanent? In Vitro 10: 97–111. 4143484 10.1007/BF02615343

[29] Friedenstein AJ, Gorskaja JF, Kulagina NN (1976) Fibroblast precursors in normal and irradiated mouse hematopoietic organs. Exp Hematol 4: 267–274. 976387

[30] Sekiya I, Larson BL, Smith JR, Pochampally R, Cui JG, et al (2002) Expansion of human adult stem cells from bone marrow stroma: conditions that maximize the yields of early progenitors and evaluate their quality. Stem Cells 20: 530–541. 12456961 10.1634/stemcells.20-6-530

[31] Simmons DJ, Seitz P, Kidder L, Klein GL, Waeltz M, et al (1991) Partial characterization of rat marrow stromal cells. Calcif Tissue Int 48: 326–334. 1647262 10.1007/BF02556152

[32] Aubin JE (1999) Osteoprogenitor cell frequency in rat bone marrow stromal populations: role for heterotypic cell-cell interactions in osteoblast differentiation. J Cell Biochem 72: 396–410. 10022521

[33] Katagiri T, Yamaguchi A, Komaki M, Abe E, Takahashi N, et al (1994) Bone morphogenetic protein-2 converts the differentiation pathway of C2C12 myoblasts into the osteoblast lineage. J Cell Biol 127: 1755–1766. 7798324 10.1083/jcb.127.6.1755 PMC2120318

[34] Chalaux E, Lopez-Rovira T, Rosa JL, Bartrons R, Ventura F (1998) JunB is involved in the inhibition of myogenic differentiation by bone morphogenetic protein-2. J Biol Chem 273: 537–543. 9417113 10.1074/jbc.273.1.537

[35] Fujii M, Takeda K, Imamura T, Aoki H, Sampath TK, et al (1999) Roles of bone morphogenetic protein type I receptors and Smad proteins in osteoblast and chondroblast differentiation. Mol Biol Cell 10: 3801–3813. 10564272 10.1091/mbc.10.11.3801 PMC25680

[36] Teboul L, Gaillard D, Staccini L, Inadera H, Amri EZ, et al (1995) Thiazolidinediones and fatty acids convert myogenic cells into adipose-like cells. J Biol Chem 270: 28183–28187. 7499310 10.1074/jbc.270.47.28183

[37] Rando TA, Blau HM (1994) Primary mouse myoblast purification, characterization, and transplantation for cell-mediated gene therapy. J Cell Biol 125: 1275–1287. 8207057 10.1083/jcb.125.6.1275 PMC2290930

[38] Wada MR, Inagawa-Ogashiwa M, Shimizu S, Yasumoto S, Hashimoto N (2002) Generation of different fates from multipotent muscle stem cells. Development 129: 2987–2995. 12050145 10.1242/dev.129.12.2987

[39] Hashimoto N, Kiyono T, Wada MR, Umeda R, Goto Y, et al (2008) Osteogenic properties of human myogenic progenitor cells. Mech Dev 125: 257–269. 18164186 10.1016/j.mod.2007.11.004

[40] Tamaki T, Okada Y, Uchiyama Y, Tono K, Masuda M, et al (2007) Clonal multipotency of skeletal muscle-derived stem cells between mesodermal and ectodermal lineage. Stem Cells 25: 2283–2290. 17588936 10.1634/stemcells.2006-0746

[41] Tamaki T, Akatsuka A, Ando K, Nakamura Y, Matsuzawa H, et al (2002) Identification of myogenic-endothelial progenitor cells in the interstitial spaces of skeletal muscle. J Cell Biol 157: 571–577. 11994315 10.1083/jcb.200112106 PMC2173851

[42] Torrente Y, Tremblay JP, Pisati F, Belicchi M, Rossi B, et al (2001) Intraarterial injection of muscle-derived CD34(+)Sca-1(+) stem cells restores dystrophin in mdx mice. J Cell Biol 152: 335–348. 11266450 10.1083/jcb.152.2.335 PMC2199616

[43] Lee JY, Qu-Petersen Z, Cao B, Kimura S, Jankowski R, et al (2000) Clonal isolation of muscle-derived cells capable of enhancing muscle regeneration and bone healing. J Cell Biol 150: 1085–1100. 10973997 10.1083/jcb.150.5.1085 PMC2175240

[44] Jackson KA, Mi T, Goodell MA (1999) Hematopoietic potential of stem cells isolated from murine skeletal muscle. Proc Natl Acad Sci U S A 96: 14482–14486. 10588731 10.1073/pnas.96.25.14482 PMC24462

[45] Gussoni E, Soneoka Y, Strickland CD, Buzney EA, Khan MK, et al (1999) Dystrophin expression in the mdx mouse restored by stem cell transplantation. Nature 401: 390–394. 10517639 10.1038/43919

[46] Morikawa S, Mabuchi Y, Kubota Y, Nagai Y, Niibe K, et al (2009) Prospective identification, isolation, and systemic transplantation of multipotent mesenchymal stem cells in murine bone marrow. J Exp Med 206: 2483–2496. 19841085 10.1084/jem.20091046 PMC2768869

[47] Baddoo M, Hill K, Wilkinson R, Gaupp D, Hughes C, et al (2003) Characterization of mesenchymal stem cells isolated from murine bone marrow by negative selection. J Cell Biochem 89: 1235–1249. 12898521 10.1002/jcb.10594

[48] Eslaminejad MB, Nikmahzar A, Taghiyar L, Nadri S, Massumi M (2006) Murine mesenchymal stem cells isolated by low density primary culture system. Dev Growth Differ 48: 361–370. 16872449 10.1111/j.1440-169X.2006.00874.x

[49] Feitosa ML, Fadel L, Beltrao-Braga PC, Wenceslau CV, Kerkis I, et al (2010) Successful transplant of mesenchymal stem cells in induced osteonecrosis of the ovine femoral head: preliminary results. Acta cirurgica brasileira/Sociedade Brasileira para Desenvolvimento Pesquisa em Cirurgia 25: 416–422. 10.1590/s0102-86502010000500006 20877951

[50] Horie M, Driscoll MD, Sampson HW, Sekiya I, Caroom CT, et al (2012) Implantation of allogenic synovial stem cells promotes meniscal regeneration in a rabbit meniscal defect model. The Journal of bone and joint surgery American volume 94: 701–712. 22517386 10.2106/JBJS.K.00176 PMC3326686

[51] Jones BA, Pei M (2012) Synovium-Derived Stem Cells: A Tissue-Specific Stem Cell for Cartilage Engineering and Regeneration. Tissue engineering Part B, Reviews. 10.1089/ten.TEB.2012.0002 22429320

[52] Vinardell T, Sheehy EJ, Buckley CT, Kelly DJ (2012) A Comparison of the Functionality and In Vivo Phenotypic Stability of Cartilaginous Tissues Engineered from Different Stem Cell Sources. Tissue engineering Part A. 10.1089/ten.tea.2011.0544 PMC3360504 22429262

[53] Wu L, Prins HJ, Helder MN, van Blitterswijk CA, Karperien M (2012) Trophic Effects of Mesenchymal Stem Cells in Chondrocyte Co-Cultures are Independent of Culture Conditions and Cell Sources. Tissue engineering Part A. 10.1089/ten.TEA.2011.0715 22429306

